# Acute myocardial infarction presenting as headache: a case report

**DOI:** 10.1186/s13256-024-04913-5

**Published:** 2024-12-02

**Authors:** Ahmed Emara, Amr K. Hassan, Ahmed M. Khairy

**Affiliations:** 1 Faculty of Medicine, Al-Azhar University, Cairo, Egypt; 2 School of Medicine, University of California, Irvine, CA USA

**Keywords:** Myocardial infarction, Headache, Atypical presentation, Case report

## Abstract

**Background:**

The diagnosis of acute myocardial infarction is straightforward when it presents with typical symptoms. However, some patients can be asymptomatic, and some can present with atypical symptoms.

**Case presentation:**

We present a very rare case of acute myocardial infraction that presented with headache and right upper limb numbness of 2-day duration in a 61-year-old female Middle Eastern patient. The patient underwent percutaneous coronary intervention to a totally occluded right coronary artery with two drug-eluting stents. The headache and numbness immediately disappeared after revascularization, and the patient was discharged on anti-ischemic medications. This report concerns a case of cardiac cephalalgia.

**Conclusion:**

Myocardial infarction can present in several different ways and should be included in the differential diagnosis for headache.

## Background

Acute myocardial infarction (AMI) is one of leading causes of death worldwide. Diagnosis of acute myocardial infarction is straightforward when it presents with typical symptoms. Most patients with acute myocardial infarction present with severe squeezing or crushing pressure pain in the sternum, often radiating to the left arm, neck, or jaw [1].The pain is like the pain of angina pectoris, but it can be differentiated by its intensity, duration (> 30 minute), and failure to respond to nitroglycerin. The pain may be associated with nausea, vomiting, and diarrhea, especially when the infarction is in the inferior wall [2].

Other symptoms include dyspnea, diaphoresis, dizziness, palpitations, cold perspiration, profound weakness, and syncope [2]. Diagnosis of AMI may be difficult when atypical symptoms occur, such as indigestion, unusual localization of the pain, agitation, and altered mental status. Some patients may be asymptomatic [1].

We describe a case of acute myocardial infarction that presented with headache and right upper limb numbness as an atypical presentation of acute myocardial infarction.

## Case report

**Fig. 1:**
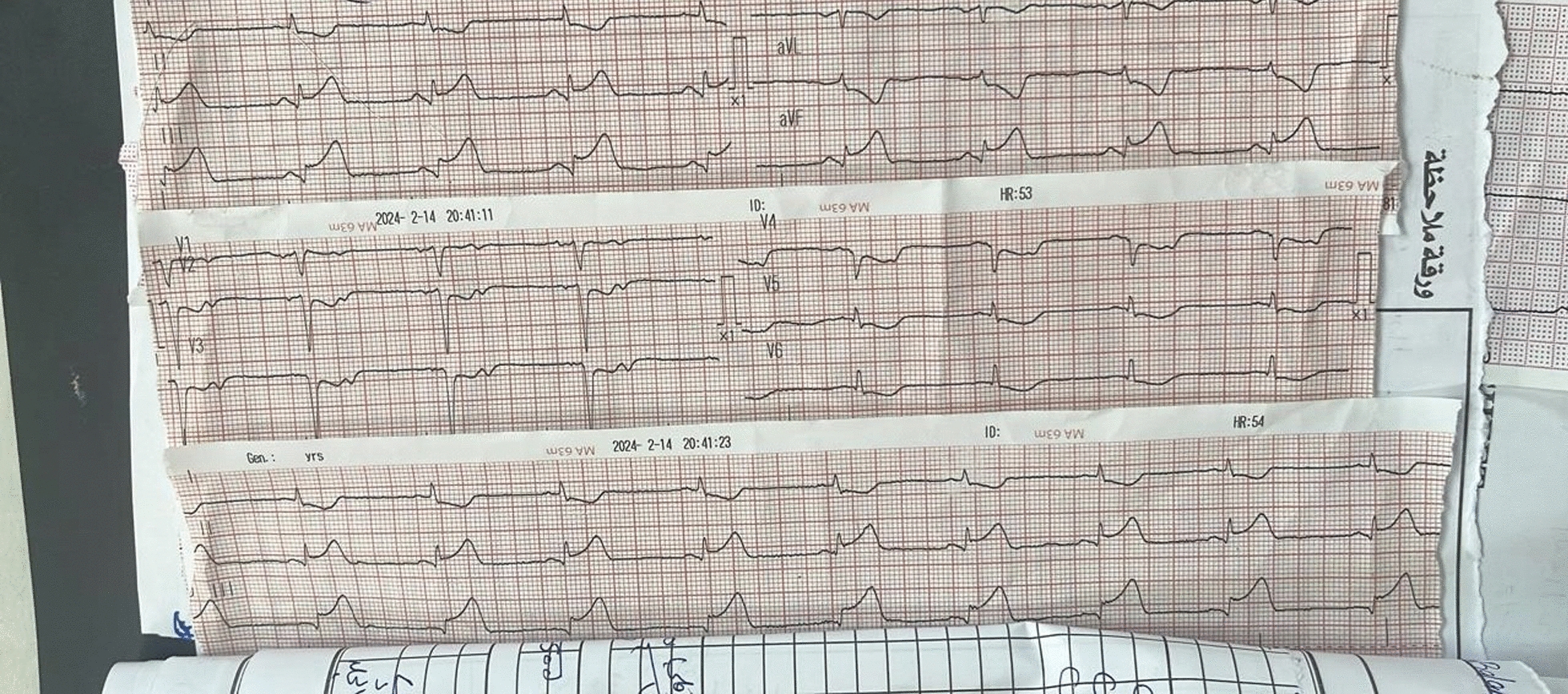
Electrocardiogram showing ST segment elevation in inferior leads (lead II, III, and aVF) with reciprocal ST segment depression in leads I and aVL

A 61-year-old female Middle Eastern patient who is known to be hypertensive but not diabetic, with no past cardiac history, presented to the emergency room, complaining of headache and right upper limb numbness of 2-day duration. She was admitted to the department of neurology under observation. Brain computed tomography (CT) was done and was normal. During routine cardiac evaluation, electrocardiogram (ECG) was done and revealed ST segment elevation in inferior leads (lead II, III, and aVF) with reciprocal ST segment depression in leads I and aVL (Fig. 1). Cardiac enzymes were done, and showed positive troponin I level and creatine kinase-MB (CKMB). On the basis of the findings of ECG and cardiac enzymes, myocardial infarction was diagnosed.

**Fig. 2:**
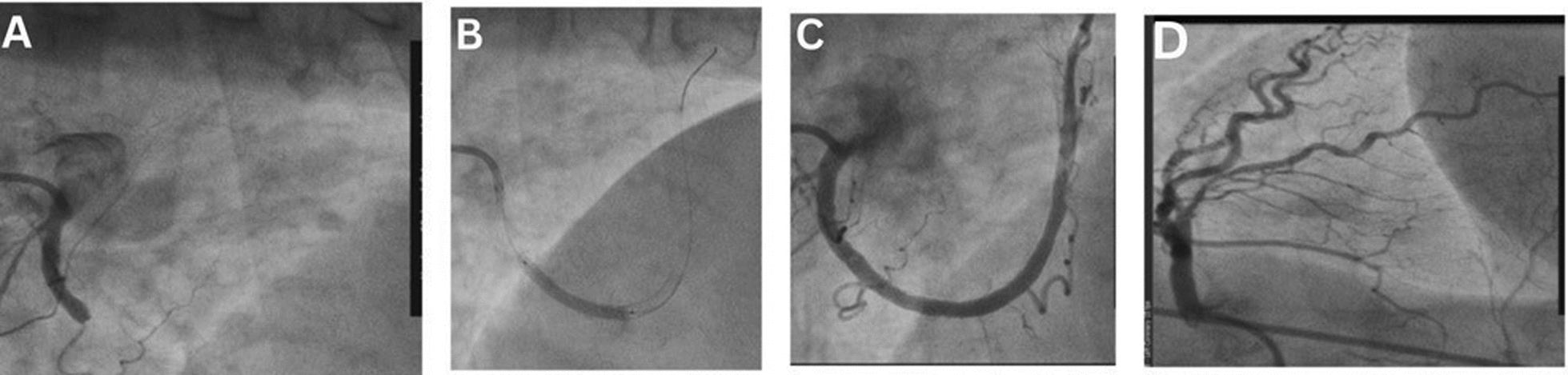
Coronary angiography showed (**A**) a normal left main coronary artery, (**B**) an atherosclerotic left anterior descending artery with mild long significant lesion, (**C**) an atherosclerotic left circumflex artery with no significant lesion, and (**D**) proximal total occlusion of the right coronary artery

The patient was treated with a loading dose of clopidogrel 600 mg, aspirin 300 mg, and heparin 5000 IU. The cath lab was activated, and coronary angiography was done to show a normal left main coronary artery, an atherosclerotic left anterior descending (LAD) artery with mild long significant lesion, an atherosclerotic left circumflex artery (LCX) with no significant lesion, and a proximal total occlusion of the right coronary artery (RCA) (Fig. 2).

**Fig. 3:**
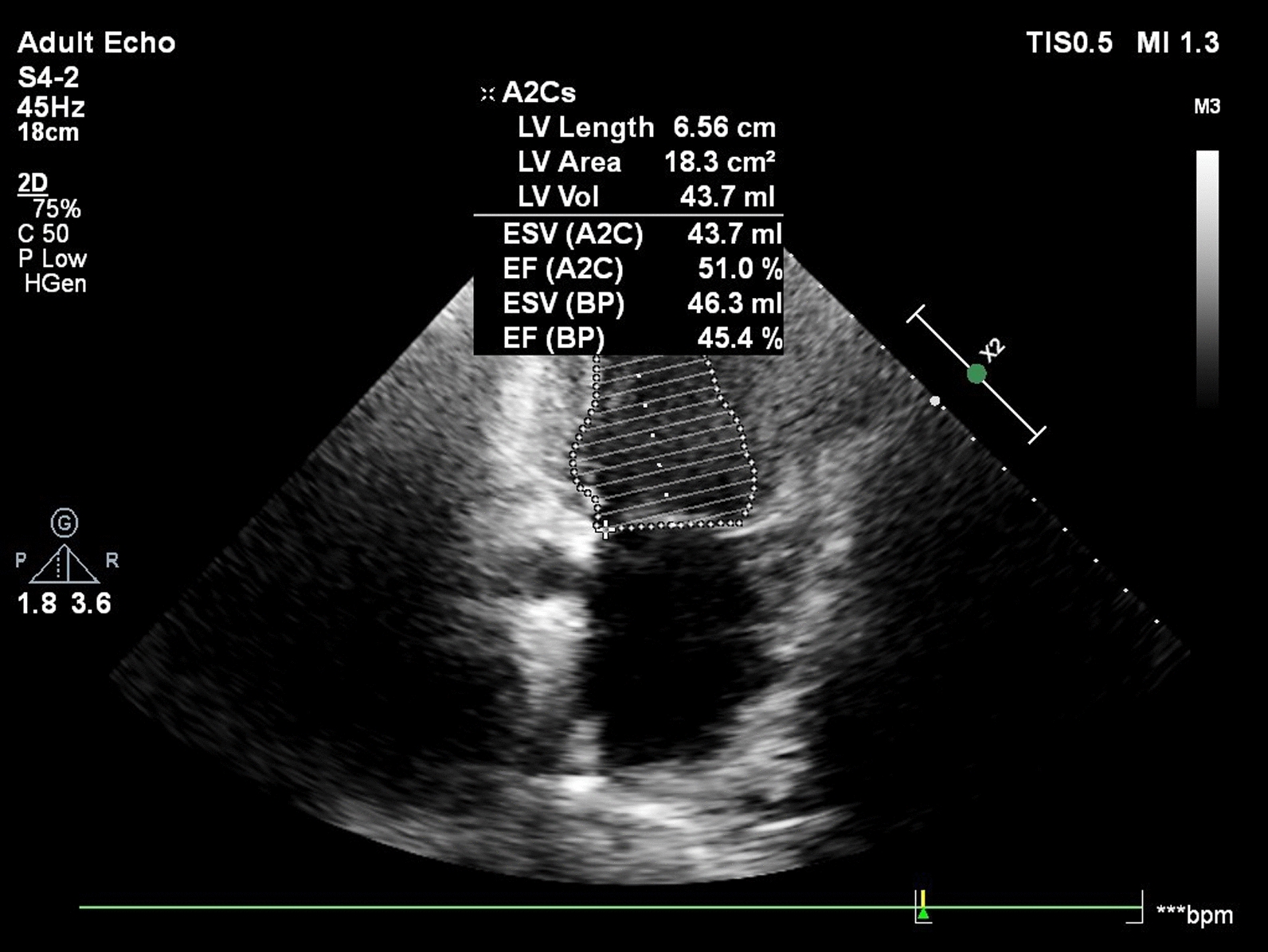
Echocardiography showing ischemic heart disease with mild impairment of the left ventricle systolic function and a hypokinetic mid, basal inferior, and inferior lateral segments

Percutaneous coronary intervention was done to the RCA with 2 drug-eluting stents, and the patient was scheduled for another session for the LAD. Echocardiography showed ischemic heart disease with mild impairment of the left ventricle systolic function (LV-EF 45%) and a hypokinetic mid, basal inferior, and inferior lateral segments (Fig. 3).

The headache and numbness completely resolved after vascularization, and the patient remained asymptomatic for the rest of the hospital stay. On follow-up ECG, the ST segment changes gradually returned to normal. The patient was discharged from hospital on anti-ischemic medications.

## Discussion and conclusions

Diagnosis of acute myocardial infarction is straightforward for most patients who present with typical symptoms. However, the diagnosis becomes very difficult when a patient presents with atypical, rare symptoms. In most cases, headache presents with other coexisting typical symptoms of acute myocardial infarction [2]. On the other hand, isolated headache with right-side numbness is a very rare presentation of acute myocardial infarction. Only a few cases are reported, relating to patients over 60 years old, diabetic, or female [3, 4]. There are many risk factors for coronary artery diseases such as hypertension, diabetes mellitus, smoking, obesity, and positive family history. So, acute myocardial infarction should be suspected and included in physicians’ minds while evaluating patients with any of these risk factors [1].

In this case report, we describe the association between headache and acute myocardial infarction, which is proved by the immediate disappearance of headache after revascularization. The underlying pathophysiology of such a headache in the case of acute myocardial infarction is still unclear but is postulated to be related to acute reduction of the cardiac output due to acute myocardial infarction, especially in the presence of cerebral artery atherosclerosis. Alternatively, a headache in the setting of an acute cardiac event may be related to a generalized catecholamine-induced vasospastic disorder and subsequent increase of intracranial pressure [5].

Another explanation of the headache in the setting of acute myocardial infarction involves the perception of cardiac ischemic pain as headache owing to the convergence—most likely at a thalamic level—of cardiac autonomic nerve fibers with somatic nerves originating from the head [3, 4, 6]. Finally, it has been suggested that headache-eliciting mediators released during cardiac ischemia may also have a role in the manifestation of headache; however, this issue is still under investigation [6].

In conclusion, we present a very rare case of acute myocardial infarction manifested with headache and upper limb numbness as atypical symptoms. For this reason, physicians should always take into consideration that headache could be a symptom of coronary artery disease. So, there is a big possibility of the incidence of ischemic heart disease in patients presenting with headache after exclusion of intracranial causes, especially for patients who present with multiple risk factors.

## Data Availability

Anonymized data from the patient are available. They can be provided upon reasonable request for known parties.
